# Loop Diuretic Therapy in Severe Aortic Stenosis: Marker of Organ Congestion, Unfavorable Hemodynamics, and Increased Post‐Valve Replacement Mortality

**DOI:** 10.1002/clc.70225

**Published:** 2025-11-29

**Authors:** Micha T. Maeder, Alexander Breuss, Sharon Appert, Simon Wildermuth, Philipp K. Haager, Johannes Rigger, Joannis Chronis, Martin O. Schmiady, Hans Rickli, Lukas Weber

**Affiliations:** ^1^ Department of Cardiology HOCH Health Ostschweiz, Kantonsspital St. Gallen St. Gallen Switzerland; ^2^ University of Basel Basel Switzerland; ^3^ Clinic of Intensive Care University Hospital Zürich Zürich Switzerland; ^4^ Department of Radiology Kantonsspital St. Gallen St. Gallen Switzerland; ^5^ Clinic of Cardiac Surgery University Hospital Zürich Switzerland; ^6^ Departmentof Cardiology and Cardio‐Vascular Surgery Hôspital Cardiologique de Haut‐Lévêque, Bordeaux University Hospital France

**Keywords:** aortic stenosis, congestion, diuretic, valve replacement

## Abstract

**Background:**

Loop diuretic therapy (LDT) is associated with increased mortality in heart failure. Severe aortic stenosis (AS) patients are at risk for heart failure and frequently on LDT. We assessed cardiac structure and function, organ congestion, filling pressures, and long‐term outcomes of severe AS patients on LDT undergoing aortic valve replacement (AVR).

**Methods:**

Consecutive patients with severe AS with [*n* = 157; median (interquartile range) daily torasemide dose: 10 (5–15) mg] or without (*n* = 346) LDT undergoing a detailed assessment of congestion (B‐type natriuretic peptide, liver enzymes, systematic chest X‐ray analysis) and cardiac catheterization before AVR with a post‐AVR follow‐up of several years were studied.

**Results:**

Despite similar AS severity (indexed aortic valve area 0.41 ± 0.12 vs. 0.43 ± 0.12 cm^2^/m^2^) patients with LDT had more advanced biventricular remodeling and dysfunction, higher B‐type natriuretic peptide [446 (245–991) vs. 150 (62–317) ng/L; *p* < 0.001], higher liver enzymes, higher chest x‐ray congestion score [2 (1–4.5) vs. 1 (0–2) score points; *p* < 0.001], and higher mean right atrial pressure (8 ± 4 vs*.* 6 ± 3 mmHg) and mean pulmonary artery wedge pressure (21 ± 8 vs. 14 ± 6 mmHg; *p* < 0.001 for both) than those without. After a median post‐AVR follow‐up of 15 months functional capacity was worse, and estimated systolic pulmonary pressure was higher (37 ± 11 vs*.* 32 ± 8 mmHg; *p* < 0.001), and after a median follow‐up of 44 months mortality was higher [hazard ratio 2.01 (95% confidence interval 1.17–3.77); *p* = 0.01] in LDT compared to non‐LDT patients.

**Conclusions:**

LDT identifies AS patients with more advanced cardiac remodeling, more severe congestion, unfavorable hemodynamics, impaired post‐AVR status, and increased post‐AVR long‐term mortality.

## Graphical abstract

Aortic stenosis patients on loop diuretic therapy have more evidence of congestion, higher filling pressures, and lower cardiac index, worse functional status several months after aortic valve replacement, and higher long‐term mortality after valve replacement than those without. Loop diuretic therapy identifies a high risk group of aortic stenosis patients. AS = aortic stenosis; AVR = aortic valve replacement, BNP = B‐type natriuretic peptide; FU = follow‐up; GGT = Gamma‐glutamyl transferase; LDT = loop diuretic therapy; mPAWP = mean pulmonary artery wedge pressure; sPAP = systolic pulmonary artery pressure.

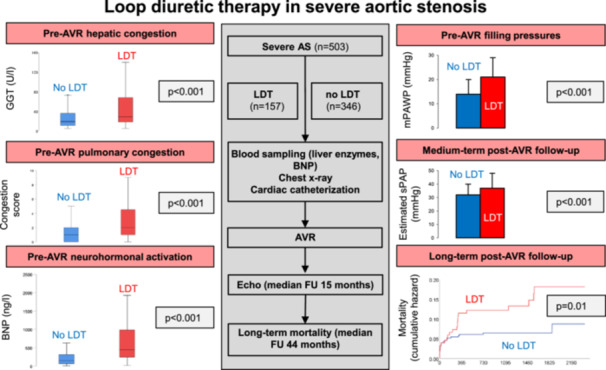

## 1 Introduction

Loop diuretic therapy (LDT) represents a mainstay in the management of multiple heart failure (HF) syndromes [1]. Although there are no data from randomized controlled trials demonstrating the efficacy of LDT to improve clinical outcomes, consistent experience from clinical practice has led to the widespread use of LDT for the reduction of congestion and symptom relief in HF, and this practice is supported by international guideline recommendations [2, 3]. However, LDT can be associated with important side effects including electrolyte disturbances, activation of the renin–angiotensin–aldosterone system, and poor tolerability of guideline‐recommended HF therapy. There is also increasing and consistent evidence that LDT is associated with increased mortality in HF patients [4, 5, 6, 7]. Importantly, LDT is associated with an increased risk of death even in subjects without an established diagnosis of HF [6]. Given the observational nature of all these data, it is currently unknown whether this association is mediated by an adverse effect of LDT, or whether LDT is a pure marker of a more advanced disease stage, or whether both apply [6].

Aortic stenosis (AS) is the most common valve disease in the elderly, which results in HF if left untreated. The occurrence of symptoms (i.e., HF) represents the main indication for aortic valve replacement (AVR) because prognosis is poor once HF symptoms have manifested [8]. However, in clinical practice, HF in AS is often first managed medically, typically by LDT. Interestingly, there is very limited data on the characteristics and outcomes of patients treated with LDT in this very common setting. Patients with AS on LDT undergoing transcatheter AVR (TAVR) were found to have more comorbidities and worse outcomes than those without LDT [9]. It has also been reported that patients with increased loop diuretic dose post‐TAVR had a higher long‐term mortality compared to those with unchanged/reduced dose [10]. The aim of the present study was to provide a detailed clinical, echocardiographic, biochemical, radiological and invasive characterization of the presence and extent of congestion and long‐term post‐AVR functional status and mortality of unselected AS patients treated with TAVR or surgical AVR (SAVR) with versus without pre‐AVR LDT.

## 2 Methods

### 2.1 Study Population

This is a retrospective analysis of prospectively and systematically collected cardiac catheterization data in a cohort of 503 consecutive patients with severe AS undergoing cardiac catheterization prior to AVR in a single center between January 2011 and January 2016 and with a post‐AVR follow‐up of several years [11]. At our institution, all patients with severe AS undergo right heart catheterization and coronary angiography on a routine basis prior to AVR. Accordingly, this is an unselected all‐comers cohort of AS patients evaluated prior to AVR. Data on LDT and the daily dose of torasemide, respectively, were available for all patients. In clinical practice, torasemide is the only oral loop diuretic used in our country. Thiazides and thiazide‐like diuretics were not counted as LDT because in our country, these drugs are typically not used to treat HF but as antihypertensive therapy, most often in combination with an angiotensin converting enzyme inhibitor (ACEI) or an angiotensin receptor blocker (ARB). The study was approved by the local ethics committee. Owing to its retrospective design, a waiver of consent was granted for this study.

### 2.2 Noninvasive Assessment of Congestion

A venous blood sample was obtained in all patients on the day prior to cardiac catheterization. Analysis was performed immediately after blood collection in the local biochemistry laboratory. The estimated glomerular filtration rate (eGFR) was calculated according to the new Chronic Kidney Disease Epidemiology Collaboration formula, which is based on serum creatinine, age, and sex [12]. The eGFR has been shown to be related to venous congestion in HF [13] and AS [14]. Aspartate transferase, alanine transferase, alkaline phosphatase, and gamma‐glutamyl transferase were measured as markers of liver damage and congestion, respectively [15]. In 300 patients, BNP was measured using a commercially available and well‐characterized fluorescence immunoassay (Biosite Triage, Biosite Inc., San Diego, CA, USA). In 471 patients, a digitally stored upright chest X‐ray (CXR) obtained prior to cardiac catheterization (in the vast majority on the day before) with sufficient image quality was available. Images were retrospectively reviewed in a systematic manner by two radiologists who were blinded to all clinical data as previously described [16]. A radiological congestion score (RxCS) was applied as described previously [16, 17]. In brief, the RxCS includes six items, including redistribution of lung vessels, enlarged cardiac silhouette, and peribronchial cuffing (each scored with 0 to 1 points), pleural effusion and Kerley's lines (each scored with 0–2 points), and lung opacity (scored with 0 to 3 points) [17]. Thus, the maximal RxCS is 10 points. The two radiologists performed an independent CXR assessment, and the RxCS used for analysis was the average of the two ratings. Thus, also half points were possible, for example, the RxCS was 1.5 if radiologist A scored one point, and radiologist B scored two points.

### 2.3 Echocardiography

In all patients, an echocardiogram was carried out prior to performing cardiac catheterization, and it was used as a basis for the referral. Echocardiograms were performed by experienced cardiologists in line with contemporary guidelines, but without adhering to a specified protocol. The data were retrospectively retrieved from patients' medical reports.

### 2.4 Cardiac Catheterization

Procedures were generally (> 95%) performed in the morning in the fasting state and after withholding loop diuretics and renin‐angiotensin system inhibitors. Patients underwent coronary angiography using 5 or 6 French catheters via the femoral or radial artery and right heart catheterization using 6 French Swan‐Ganz catheters via femoral or brachial access. The midthoracic level was used as zero reference point. Right atrial pressure, right ventricular pressure, pulmonary artery pressure (PAP), and pulmonary artery wedge pressure were measured. The wedge position was confirmed by fluoroscopy and waveform analysis. Measurements were obtained at end‐expiration, the mean pulmonary artery wedge pressure (mPAWP) was calculated over the entire cardiac cycle, and v waves were included. This practice leads to higher values compared to the measurement of the end‐diastolic pulmonary artery wedge pressure. However, for the estimation of the impact of the left heart contribution to pulmonary pressures and calculation of pulmonary vascular resistance (PVR), respectively, the mPAWP is preferred [18]. In patients with atrial fibrillation, at least five cardiac cycles were used to assess pulmonary artery pressure and pulmonary artery wedge pressure. Cardiac output was assessed by the indirect Fick method based on blood gases, with blood samples taken in duplicate via arterial access and pulmonary artery. The transpulmonary gradient was calculated as the difference between mean PAP (mPAP) and mPAWP, and PVR as transpulmonary gradient divided by cardiac output. The pulmonary artery capacitance (PAC) was calculated as stroke volume divided by the pulmonary artery pulse pressure (i.e., difference between systolic and diastolic PAP). If the aortic valve was crossed, the LV end‐diastolic pressure (LVEDP) was recorded. All pressure readings were double‐checked by the operator using manual review of the pressure tracings before recording them into the report.

### 2.5 Follow‐Up

All patients underwent surgical AVR (SAVR; *n* = 361; 72%) or TAVR (*n* = 142; 28%) following a median interval of 21 (12–35) days post‐catheterization. Information on follow‐up examinations (symptoms, echocardiography, exercise testing) and clinical long‐term follow‐up (endpoint all‐cause mortality) was collected manually by a research assistant from patients, general practitioners, and hospital or practice cardiologists.

### 2.6 Statistical Analysis

Categorical data are presented as numbers and percentages, and continuous data as mean ± standard deviation or median (interquartile range; IQR) as appropriate. Patients with and without LDT were compared using unpaired *t*‐tests, Mann–Whitney *U* test, or chi‐square tests as appropriate. For the comparison of hemodynamics in patients with high‐dose LDT (daily torasemide dose > 10 mg), low‐dose LDT (daily torasemide dose ≤ 10 mg), and no LDT, as well as patients in different LDT/mPAWP strata (for definition see below) analysis of variance was applied. Correlations between the daily torasemide dose (skewed distribution) and hemodynamics were expressed by Spearman correlation coefficients. Survival of patients with versus without LDT and LDT categories, respectively, was investigated using Kaplan Meier estimates and compared using log‐rank tests. Cox proportional hazards regression was applied to describe the association between LDT and LDT categories respectively and mortality, where LDT was tested both as categorical (any LDT, high‐dose and low‐dose) and continuous (daily torasemide dose) variable. A *p* < 0.05 was considered statistically significant. No adjustment for multiple testing was performed. Analyses were performed using SPSS statistical package Version 20.0 (SPSS Inc., Chicago, IL, USA).

## 3 Results

### 3.1 Study Population

The mean age of the population was 74 ± 10 years, and 290/503 (58%) were male. The mean indexed aortic valve area was 0.42 ± 0.12 cm^2^/m^2^, and the mean left ventricular (LV) ejection fraction (LVEF) was 57% ± 12%. Among the 503 patients, 157 (31%) patients were on LDT. The median (IQR) daily torasemide dose in these patients was 10 (5–15) mg.

### 3.2 Clinical Characteristics of Patients With Versus Without LDT

Patients on LDT were older and more likely to have atrial fibrillation and a history of a previous stroke compared to those not on LDT. Patients with LDT were also more likely to use oral anticoagulation, betablockers, spironolactone, and digoxin, but were less likely to use thiazides compared to those without LDT. There was no difference in terms of ACEI/ARB use between groups. Patients on LDT were more symptomatic and had higher surgical risk based on standard scores (Table 1).

**Table 1:** Clinical characteristics of patients with versus without loop diuretic therapy (LDT).

	LDT (*n* = 157)	No LDT (*n* = 346)	*p*
Age (years)	78 ± 9	73 ± 10	< 0.001
Sex (male)	83 (53%)	207 (60%)	0.14
Body mass index (kg/m^2^)	28.1 ± 5.4	27.8 ± 5.0	0.54
Diabetes	38 (24%)	67 (19%)	0.22
Insulin‐dependent	8 (1%)	14 (1%)	0.59
Previous stroke	15 (10%)	15 (4%)	0.02
Chronic obstructive pulmonary disease	17 (11%)	42 (12%)	0.67
Previous percutaneous coronary intervention	17 (11%)	29 (8%)	0.38
Previous coronary artery bypass grafting	11 (7%)	16 (5%)	0.27
Heart rhythm^a^			< 0.001
Sinus rhythm	116 (74%)	314 (91%)	
Atrial fibrillation	31 (20%)	17 (5%)	
Pacemaker	8 (5%)	12 (3%)	
Other rhythm	2 (1%)	3 (1%)	
Medication			
Oral anticoagulation	45 (29%)	49 (17%)	< 0.001
Aspirin	88 (56%)	220 (%)	0.11
Loop diuretics	157 (100%)	–	
Torasemide dose (mg)	10 (5–15)	–	
Thiazide diuretic	0 (%)	92 (27%)	< 0.001
Spironolactone	17 (11%)	8 (2%)	< 0.001
Betablocker	85 (54%)	153 (44%)	0.04
ACEI/ARB	84 (54%)	193 (45%)	0.63
Digoxin	26 (17%)	6 (2%)	< 0.001
Symptoms			
Dyspnea NYHA class			< 0.001
I	17 (11%)	84 (24%)	
II	57 (36%)	189 (55%)	
III	67 (43%)	66 (19%)	
IV	16 (10%)	7 (2%)	
STS score	3.2 (2.3–5.1)	2.2 (1.3–3.1)	< 0.001
Logistic EuroScore	4.0 (2.2–5.6)	2.6 (1.4–3.6)	< 0.001
Mode of AVR			< 0.001
Surgical AVR	84 (54%)	277 (80%)	
Transcatheter AVR	73 (46%)	69 (20%)	

*Note:* Data are given as numbers and percentages, mean ± standard deviation, or median (interquartile range).

Abbreviations: ACEI/ARB, angiotensin converting enzyme inhibitor/angiotensin receptor blocker; AVR, aortic valve replacement; NYHA, New York Heart Association; STS, Society of Thoracic Surgeons.

a Rhythm at the time of cardiac catheterization.

### 3.3 Cardiac Structure and Function of Patients With Versus Without LDT

Patients on LDT had larger LV size, worse LVEF and LV diastolic function, larger left atrial size, more severe mitral regurgitation, larger right ventricular and right atrial dimensions, worse right ventricular function, and higher estimated systolic PAP compared to patients without LDT. The indexed aortic valve area did not significantly differ between groups, however (Table 2).

**Table 2:** Data from echocardiography and cardiac catheterization of patients with versus without loop diuretic therapy (LDT).

	LDT (*n* = 157)	No LDT (*n* = 346)	*p*
Echocardiography			
Indexed left ventricular end‐diastolic diameter (mm/m^2^)	26 ± 5	24 ± 4	< 0.001
Septal wall thickness (mm)	13 ± 3	13 ± 3	0.20
Posterior wall thickness (mm)	11 ± 3	11 ± 3	0.41
Left ventricular mass index (g/m^2^)	121 ± 40	106 ± 32	< 0.001
Left ventricular ejection fraction (%)	52 ± 14	60 ± 10	< 0.001
E/e′	20 ± 10	15 ± 7	< 0.001
Indexed left atrial diameter (mm/m^2^)	23 ± 4	21 ± 4	< 0.001
Indexed left atrial area (cm^2^/m^2^)	14 ± 4	12 ± 3	< 0.001
Left atrial volume index (ml/m^2^)	49 ± 20	39 ± 14	< 0.001
Indexed right ventricular basal diameter (mm/m^2^)	17 ± 4	16 ± 3	0.001
Indexed right atrial area (cm^2^/m^2^)	10 ± 3	8 ± 2	< 0.001
Right atrial volume index (ml/m^2^)	26 ± 12	22 ± 10	< 0.001
Tricuspid annular plane systolic excursion (mm)	20 ± 5	22 ± 5	< 0.001
Estimated systolic pulmonary artery pressure (mmHg)	46 ± 13	36 ± 12	< 0.001
Mean aortic valve gradient (mmHg)	46 ± 19	48 ± 17	0.34
Peak aortic valve gradient (mmHg)	71 ± 26	77 ± 24	0.02
Aortic valve area (cm^2^)	0.76 ± 0.23	0.80 ± 0.24	0.09
Indexed aortic valve area (cm^2^/m^2^)	0.41 ± 0.12	0.43 ± 0.12	0.09
Mitral regurgitation			< 0.001
no	42 (27%)	196 (56%)	
mild	88 (56%)	127 (37%)	
moderate	23 (15%)	17 (5%)	
severe	4 (2%)	6 (2%)	
Coronary angiography			0.50
No coronary artery disease	82 (52%)	182 (53%)	
1‐vessel disease	22 (14%)	65 (19%)	
2‐vessel disease	24 (15%)	46 (13%)	
3‐vessel disease	29 (19%)	53 (15%)	
Invasive hemodynamics			
Mean aortic pressure (mmHg)	96 ± 15	99 ± 14	0.004
Systolic aortic pressure (mmHg)	141 ± 28	147 ± 24	0.01
Diastolic aortic pressure (mmHg)	65 ± 13	69 ± 11	< 0.001
Heart rate (bpm)	73 ± 14	68 ± 12	< 0.001
Mean right atrial pressure (mmHg)	8 ± 4	6 ± 3	< 0.001
Right ventricular end‐diastolic pressure (mmHg)	10 ± 4	8 ± 4	< 0.001
Systolic pulmonary artery pressure (mmHg)	49 ± 16	36 ± 12	< 0.001
Diastolic pulmonary artery pressure (mmHg)	20 ± 8	13 ± 6	< 0.001
Mean pulmonary artery pressure (mmHg)	32 ± 10	22 ± 8	< 0.001
Mean pulmonary artery wedge pressure (mmHg)	21 ± 8	14 ± 6	< 0.001
Transpulmonary gradient (mmHg)	11 ± 5	9 ± 5	< 0.001
Pulmonary vascular resistance (Wood units)	2.6 ± 1.6	1.9 ± 1.4	< 0.001
Pulmonary artery capacitance (ml/mmHg)	2.5 ± 1.4	3.7 ± 1.8	< 0.001
Left ventricular end‐diastolic pressure (mmHg) (*n* = 335)	23 ± 8	20 ± 7	0.001
Systemic vascular resistance (Wood units)	20.7 ± 5.6	19.8 ± 4.5	0.06
Arterial oxygen saturation (%)	95 (93‐96)	95 (94‐97)	< 0.001
Mixed venous oxygen saturation (%)	65 (59‐69)	70 (66‐73)	< 0.001
Cardiac output (L/min)	4.4 ± 1.1	4.9 ± 0.9	< 0.001
Cardiac index (L/min/m^2^)	2.5 ± 0.4	2.6 ± 0.5	< 0.001
Stroke volume (mL)	63 ± 20	74 ± 18	< 0.001
Stroke volume index (mL/m^2^)	34 ± 10	39 ± 9	< 0.001

*Note:* Data are given as numbers and percentages, mean ± standard deviation or median (interquartile range).

Abbreviation: E/e′, ratio of peak early mitral inflow velocity to peak early mitral annular velocity.

### 3.4 Radiological and Biochemical Evidence of Congestion in Patients With Versus Without LDT

**Figure 1:**
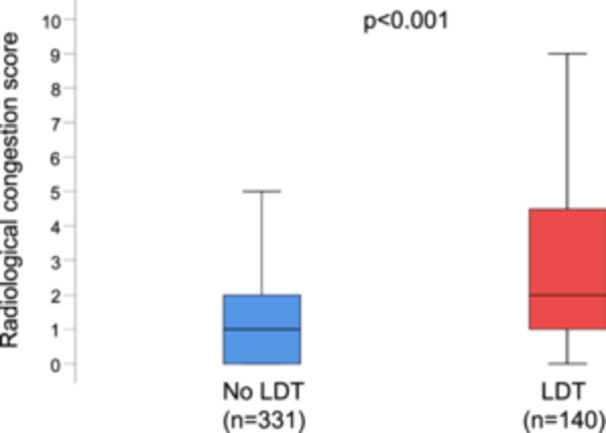
Box plots representing the radiological congestion score (RxCs) in patients with versus without loop diuretic therapy (LDT). Boxes represent medians with 25th and 75th percentiles, and whiskers represent the minima and maxima.

The RxCS was significantly higher in patients with LDT compared to those without [2 (1–4.5) vs. 1 (0–2) score points; *p* < 0.001 (Figure 1)]. B‐type natriuretic peptide was also higher in LDT patients compared to non‐LDT patients (Table 3). Patients on LDT had worse renal function as indicated by lower eGFR and higher blood urea nitrogen, and more extensive liver damage as reflected by higher aspartate transferase, alkaline phosphatase, and gamma‐glutamyl transferase than those not on LDT (Table 3).

**Table 3:** Biochemical characteristics of patients with versus without loop diuretic therapy (LDT).

	LDT (*n* = 157)	No LDT (*n* = 296)	*p*
Sodium (mmol/L)	137 ± 3	138 ± 3	0.67
Potassium (mmol/L)	4.0 ± 0.5	4.0 ± 0.5	0.67
eGFR (mL/min/1.73 m^2^)	59 ± 20	69 ± 17	< 0.001
Blood urea nitrogen (mmol/L)	8.5 ± 5.8	6.6 ± 5.4	< 0.001
Hemoglobin (g/L)	132 ± 18	136 ± 18	0.01
Albumin (g/L)	37 ± 3	39 ± 5	< 0.001
Aspartate transferase (U/L)	24 (20–32)	21 (17–26)	< 0.001
Alanine transferase (U/L)	19 (12–32)	17 (12–24)	0.15
Alkaline phosphatase (U/L)	64 (51–83)	59 (50–71)	0.001
Gamma‐glutamyl transferase (U/L)	29 (18–68)	19 (11–36)	< 0.001
B‐type natriuretic peptide (ng/L)	446 (245–991)	150 (62–317)	< 0.001
	(*n* = 104)	(*n* = 196)	

*Note:* Data are given as mean ± standard deviation or median (interquartile range).

Abbreviation: eGFR, estimated glomerular filtration rate.

### 3.5 Invasive Evidence of Congestion in Patients with Versus Without LDT

Patients on LDT had higher mean right atrial pressure (mRAP), right ventricular end‐diastolic pressure, mPAP, mPAWP, and LVEDP compared to patients not on LDT. In addition, PVR was higher, and PAC, cardiac index, and stroke volume index (SVI) were lower in LDT versus non‐LDT patients (Table 2). There were paradoxically positive correlation between the daily torasemide dose and mRAP (*r* = 0.30; *p* < 0.001), mPAP (*r* = 0.45; *p* < 0.001), mPAWP (*r* = 0.43; *p* < 0.001), and LVEDP (*r* = 0.17; *p* = 0.002).

**Figure 2:**
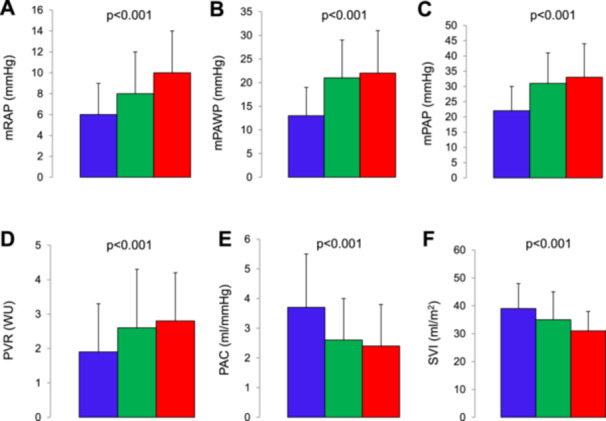
Error bars (representing mean and standard deviation) for key hemodymamic parameters in patients with high‐dose loop diuretic therapy (red), low‐dose diuretic therapy (green), and no loop diuretic therapy. (A) mRAP, mean right atrial pressure. (B) mPAWP, mean pulmonary artery wedge pressure. (C) mPAP, mean pulmonary pressure. (D) PVR, pulmonary vascular resistance. (E) PAC, pulmonary artery capacitance. (F) SVI, stroke volume index.

Hemodynamics of patients with high‐dose LDT [*n* = 46; median dose 20 (20–30) mg], low‐dose LDT [*n* = 112; median dose 10 (5–10 mg)], and no LDT are compared in Figure 2. There was evidence of a dose‐effect relationship as mRAP, mPAWP, mPAP, and PVR were highest, and PAC and SVI were lowest in the high‐dose LDT group, while values were intermediate in the low‐dose LDT group.

The majority (114/157; 73%) of patients on LDT still had an mPAWP > 15 mmHg (LDT+/PAWP+ ), while 43/157 (27%) had an mPAWP ≤ 15 mmHg (LDT+/PAWP−). On the other hand, approximately one‐third (107/346; 31%) of patients without LDT also had an mPAWP > 15 mmHg (LDT−/PAWP+), whereas in the majority of patients without LDT (239/346; 69%) the mPAWP was not elevated (LDT−/PAWP−). In Table S1, echocardiographic and invasive characteristics of patients in these four LDT/PAWP strata are compared. The LDT+ /PAWP+ group clearly had the most severe AS, the lowest LVEF, the largest left atrial dimensions, and the worst hemodynamics. The daily torasemide dose between the LDT+ /PAWP+ and the LDT+ /PAWP‐ groups did not differ [10 (5–15 mg) vs. 10 (5–15) mg; *p* = 0.59] however. The LDT−/PAWP− group had the most favorable hemodynamic constellation. The LDT +/PAWP− and LDT−/PAWP+ groups had intermediate biventricular structure and function, differed in terms of filling pressures (by definition) but had similar PVR and PAC (Table S1).

### 3.6 Long‐Term Follow of Functional Capacity and Cardiac Function in Patients /With Versus Without LDT

For 421 patients any follow‐up examination was performed after 15 (12–17) months (Table S2). Patients on LDT prior to AVR were still more symptomatic and had lower functional capacity (in absolute terms; borderline significance when expressed as percent predicted) than non‐LDT patients. In LDT patients, there was a trend towards a lower LVEF, LV diastolic function was worse, left atrial size was larger, mitral regurgitation was more severe, and the estimated systolic PAP was still higher (37 ± 11 vs*.* 37 ± 11 mmHg; *p* < 0.001) than in non‐LDT patients, while the gradient across the aortic valve prosthesis was similar in the two groups.

### 3.7 Long‐Term Mortality of Patients With Versus Without LDT

**Figure 3:**
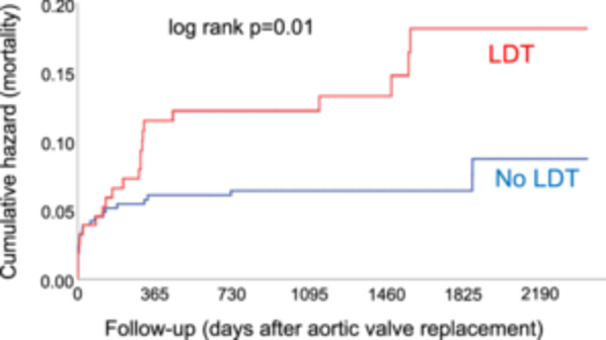
Kaplan Meier plots (cumulative hazard) comparing survival of patients with (red) versus without (blue) loop diuretic therapy (LDT).

After a median post‐AVR follow‐up of 44 (31–62) months, 45 (9%) deaths had occurred. Patients on pre‐AVR LDT had a twice as high mortality as patients not on LDT [Figure 3; hazard ratio (HR) 2.01 (95% confidence interval [95% CI] 1.17–3.77); *p* = 0.01]. Patients on high‐dose LDT had the highest mortality [HR 2.58 (95% CI 1.16–5.78); *p* = 0.02 compared to no LDT], while mortality was intermediate in low‐dose LDT patients [HR 1.87 (95% CI 0.96–3.64); *p* = 0.06 compared to no LDT] (Figure S1). When expressed as a continuous variable, every 5 mg increase in the daily torasemide dose was associated with a 5% higher mortality risk [HR 1.05 (95% 1.01–1.09); *p* = 0.01 per 5 mg increase]. In the multivariate analysis including noninvasive parameters and established surgical risk scores, the daily torasemide dose was not an independent predictor of death, however (Table S3).

The LDT + /PAWP+ group had the highest mortality [HR 2.89 (95% CI 1.42–5.90); *p* = 0.004 compared to LDT‐/PAWP‐ patients (referent)], while mortality in LDT‐/PAWP+ patients [HR 1.62 (95% CI 0.71–3.70); *p* = 0.25] and LDT+/PAWP− patients [HR 1.59 (95% CI 0.52–4.89); *p* = 0.41] was intermediate and not significantly higher than in the LDT−/PAWP− (referent) group (Figure S2).

## 4 Discussion

In this study characterizing AS patients on LDT by a comprehensive noninvasive and invasive assessment and a long‐term follow‐up over several years post‐AVR, we obtained the following key findings: First, LDT patients were older and had more co‐morbidities and higher surgical risk. Second, LDT patients had more advanced biventricular remodeling and dysfunction compared to non‐LDT patients, although the groups did not differ in terms of AS severity. Third, LDT patients had consistently more evidence of congestion (BNP, lung, liver). Fourth, filling pressures were substantially higher in LDT versus non‐LDT patients, and there were paradoxically positive correlations between the daily torasemide dose and filling pressures. Finally, LDT patients had a persistently worse status (clinical, echocardiography) several months post‐AVR and higher long‐term mortality (several years post‐AVR; central illustration).

The present study corroborates and substantially extends the findings of the study by Cantey et al. [9], which to the best of our knowledge represents the only available study systematically looking at the role of LDT in AS. In a pure TAVR population, these authors found that 48% were on LDT (mean furosemide dose equivalent of 51 mg), and that these patients were more frail and had more co‐morbidities, and more advanced cardiac remodeling. In this regard, their findings [9] are fully in line with ours but we studied an overall younger and less selected AS population with significantly lower daily loop diuretic dose (median furosemide equivalent dose in our patients approximately 20 mg). As important novel aspects compared to previous work we report a systematic assessment of organ congestion and detailed invasive hemodynamics. Notably, to assess both lung congestion and PAWP was not redundant but complimentary because it had previously shown that the correlation between the RxCs and the mPAWP is weak, and that both parameters provide independent prognostic information [16]. We found substantial differences in the extent of congestion assessed by several modalities and invasively assessed filling pressures between LDT and non‐LDT patients, and there were paradoxically positive correlations between the daily torasemide dose and filling pressures, i.e., filling pressures were high despite LDT. In accordance with a previous study [9], we found a higher proportion of patients with atrial fibrillation in the LDT group. We had previously shown that AS patients with atrial fibrillation have worse hemodynamics than those in sinus rhythm with higher mRAP and mPAWP [19].

For experienced clinicians, the present findings may not be completely surprising from a qualitative point of view. However, the magnitude and the consistency of the differences in several measures of congestion between LDT and non‐LDT patients seem impressive when considering the relatively moderate median daily torasemide dose of 10 mg in the LDT group. Only in a minority of LDT patients, filling pressure was low/normal under this therapy. Prognosis in this subgroup of LDT patients (i.e., the LDT+/PAWP− patients) was similar as in patients without LDT, whereas the majority of LDT patients had high filling pressures (i.e., the LDT+ /PAWP+ patients) despite a similar daily torasemide dose as the LDT + /PAWP− patients. It may be speculated that more aggressive LDT in the LDT+ /PAWP+ patients would have improved hemodynamics and also prognosis. The data from the present observational study do not allow definite conclusion on this aspect. However, the LDT+ /PAWP+ group was in more advanced AS stage in terms of cardiac remodeling and dysfunction, and it seems unlikely that even more aggressive LDT could have changed this substantially. At the same time, it remains unknown, whether it would be safe and maybe even beneficial to withdraw diuretics at least in selected stable and clinically euvolemic patients with AS, similarly to what has been shown for stable outpatients with heart failure with reduced ejection fraction [20]. Thus, a clinical message derived from our study could be as follows: an AS patient on LDT most often is in an advanced disease stage and may require a more urgent assessment and treatment than an AS patient not on LDT. Information on LDT use can easily be derived from a referral letter and should alert the person in charge of the triage of these patients.

It is important to realize that even AVR most often could not completely cure the disease in a stage where LDT was already required. Cantey et al. [9] reported persistent differences in cardiac structure and function 30 days post‐TAVR with higher LV mass, lower LVEF, and higher estimated systolic PAP in LDT patients. In the present study, we found a similar constellation but after a median post‐AVR follow‐up of more than 1 year. Thus, in the majority of AS patients on LDT before AVR unloading of the left ventricle did not lead to complete reversal of “cardiac damage”. We acknowledge that our technical follow‐up data are incomplete, but overall, there is a consistent picture, and it has to be taken into account that patients with such a follow‐up represented a positive selection since the sickest patients had already died at that time. The present data are overall well in line with recent large studies corroborating the important impact of the pre‐AVR cardiac damage on post‐AVR outcomes [21, 22].

In accordance with several studies among patients with HF [4, 5, 6, 7] and the only available study in AS patients [9] (mean follow‐up of 2.7 years), we found a higher all‐cause mortality in the LDT patients after a median follow‐up of 3.7 years in the unadjusted analysis. This was consistent when using LDT as a categorical or continuous variable, and there was a clear dose‐effect relationship. In a multivariate analysis including very comprehensive and established surgical risk scores, the daily torasemide dose was not an independent predictor of mortality, however. In our view, this is not a negative finding, but it highlights that LDT is a powerful marker of an advanced AS disease stage associated with a two‐fold long‐term post‐AVR mortality compared to non‐LDT patients, and therefore LDT may represent a “red flag” in AS patients. Interestingly, a recent population‐based study from Scotland found that many patients were prescribed LDT without an established diagnosis of HF, and that the mortality of these subjects was increased not only compared to subjects without HF but also compared to those with HF but no LDT [6]. It was suspected that many of these patients on LDT had unrecognized HF, and it was proposed that in any patient on LDT without an obvious indication for this therapy, a thorough review of medical records should be performed to ensure that appropriate investigations had been done to detect a serious cardiovascular pathology [6]. Similarly, LDT in an AS patient should trigger a thorough evaluation of the patient' status and the need for AVR.

It remains unknown whether LDT is also causally linked to mortality, given the potential risks associated with this therapy. However, given that the LDT + /PAWP+ and LDT + /PAWP− groups had a similar daily torasemide dose but a substantially different mortality, we assume that LDT was mainly a marker rather than a mediator of a poor prognosis. In this context, we observed lower hemoglobin and higher aspartate transaminase in patients on LDT compared to those without. Interestingly, this constellation has previously been linked to subclinical hemolysis in pre‐TAVR patients, and this in turn was found to reflect a higher procedural risk [23]. Although LDT is likely mainly a marker rather than a mediator of a higher mortality risk, a residual risk mediated by LDT cannot be excluded. Therefore, even if pre‐AVR diuretic withdrawal may not be suitable in the majority of patients (i.e., LDT + /PAWP+ patients), a switch from LDT to dapagliflozin after AVR may be a very promising strategy when considering the data from the DapaTAVI trial [24], particularly for those who were very symptomatic pre‐AVR [25].

### 4.1 Limitations

In addition to the already mentioned limitations, some additional aspects need attention. First, the number of patients and the number of events were relatively small, and therefore, power was limited. In addition, no adjustment for multiple testing was performed. Still, this is one of the largest AS cohorts reported in the literature with detailed invasive hemodynamics, and this was combined with a relatively unique systematic assessment of congestion. Second, to assess cardiac output, we have employed the indirect Fick method, which may be subject to error, as oxygen consumption is often inaccurately estimated [26]. This likely affects all cardiac output‐based measurements. It must, however, be noted that this technique is routinely used in clinical practice, and it did not affect the assessment of filling pressures, which were at the center of the present analysis. Finally, there is no detailed information about medical therapy post‐AVR. However, the focus of the study was on the pre‐AVR assessment to highlight the importance of the “LDT AS patient” as a high‐risk situation.

## 5 Conclusions

In unselected AS patients, LDT identifies those with more advanced cardiac remodeling, more severe congestion, unfavorable hemodynamics, and impaired post‐AVR status, as well as increased post‐AVR long‐term mortality. Thus, LDT is an easily available marker (e.g., from referral letters) of an advanced disease stage in AS patients, which should alert clinicians.

## Ethics Statement

The study was approved by the Local Ethics Committee.

## Consent

Owing to its retrospective design, a waiver of consent was granted for this study.

## Conflicts of Interest

The authors declare no conflicts of interest.

## Supporting information


**Supplemental Table S1:** Data from echocardiography and cardiac catheterization of patients in different loop diuretic therapy (LDT) and mean pulmonary artery wedge pressure (PAWP) strata. For definitions please see text. **Table S2:** Follow‐up data of patients with versus without loop diuretic therapy (LDT) (*n* = 421). **Supplemental Table S3:** Univariate and multivariate Cox regression analysis for the prediction of long‐term mortality after aortic valve replacement. **Supplemental Figure S1:** Kaplan Meier plots (cumulative hazard) comparing survival of patients with high‐dose loop diuretic therapy (LDT; red), low‐dose LDT (green) and no LDT (blue). **Supplemental Figure S2:** Kaplan Meier plots (cumulative hazard) comparing survival of patients in different loop diuretic use (LDT) and pulmonary artery wedge pressure (PAWP) strata. For definitions please see text.

## Data Availability

The data underlying this article will be shared on reasonable request to the corresponding author.
