# Supplementary material for: Loop Diuretic Therapy in Severe Aortic Stenosis: Marker of Organ Congestion, Unfavorable Hemodynamics, and Increased Post‐Valve Replacement Mortality
Source: Clin Cardiol. 2025 Nov 29;48(12):e70225. doi: 10.1002/clc.70225 (PMC12663767; doi:10.1002/clc.70225)

Supplemental Material

Loop diuretic therapy in severe aortic stenosis: marker of organ congestion, unfavorable hemodynamics, and increased post-valve replacement mortality

Supplemental Table S1
Supplemental Table S2
Supplemental Table S3
Supplemental Figure S1
Supplemental Figure S2

Supplemental Table S1. Data from echocardiography and cardiac catheterization of of patients in different loop diuretic therapy (LDT) and mean pulmonary artery wedge pressure (PAWP) strata. For definitions please see text.

	**LDT+/PAWP+** (n=114)	**LDT+/PAWP-** (n=43)	**LDT-/PAWP+** (n=107)	**LDT-/PAWP-** (n=239)	**P value**
**Echocardiography**					
Indexed left ventricular end-diastolic diameter (mm/m^2)	26±4	26±6	25±3	24±4	0.002
Septal wall thickness (mm)	13±3	12±3	13±3	12±3	0.08
Posterior wall thickness (mm)	11±3	11±2	11±3	11±2	0.17
Left ventricular mass index (g/m^2)	124±37	113±44	117±33	101±30	<0.001
Left ventricular ejection fraction (%)	50±15	58±10	57±11	61±9	<0.001
E/e’	22±11	17±7	16±6	15±7	<0.001
Indexed left atrial diameter (mm/m^2)	24±4	22±4	22±3	21±4	<0.001
Indexed left atrial area (cm^2/m^2)	14±4	12±3	13±3	11±3	<0.001
Left atrial volume index (ml/m^2)	51±21	41±16	46±14	37±7	<0.001
Indexed right ventricular basal diameter (mm/m^2)	18±4	16±4	16±3	16±4	0.001
Indexed right atrial area (cm^2/m^2)	10±4	9±3	9±2	8±3	<0.001
Right atrial volume index (ml/m^2)	29±17	21±10	24±9	21±10	<0.001
Tricuspid annular plane systolic excursion (mm)	19±5	22±5	22±4	23±5	<0.001
Estimated systolic pulmonary artery pressure (mmHg)	47±13	41±12	41±13	34±11	<0.001
Mean aortic valve gradient (mmHg)	46±20	46±15	48±17	48±16	0.76
Peak aortic valve gradient (mmHg)	71±28	71±23	78±26	76±24	0.14
Aortic valve area (cm^2)	0.74±0.23	0.84±0.21	0.76±0.20	0.82±0.25	0.004
Indexed aortic valve area (cm^2/m^2)	0.40±0.13	0.44±0.10	0.39±0.10	0.44±0.13	<0.001
Mitral regurgitation					<0.001
no	21 (18%)	21 (49%)	40 (37%)	156 (65%)	
mild	68 (60%)	20 (47%)	57 (53%)	70 (29%)	
moderate	21 (18%)	2 (4%)	8 (8%)	9 (4%)	
severe	4 (4%)	0 (%)	2 (2%)	4 (2%)	
**Coronary angiography**					0.82
No coronary artery disease	58 (51%)	24 (56%)	54 (50%)	128 (54%)	
1-vessel disease	17 (15%)	5 (12%)	17 (16%)	48 (20%)	
2-vessel disease	17 (15%)	7 (16%)	17 (16%)	29 (12%)	
3-vessel disease	22 (19%)	7 (16%)	19 (18%)	34 (14%)	
**Invasive hemodynamics**					
Mean aortic pressure (mmHg)	96±16	95±13	103±14	98±13	0.001
Systolic aortic pressure (mmHg)	140±29	143±25	153±28	145±22	0.004
Diastolic aortic pressure (mmHg)	66±14	69±11	71±10	69±11	0.001
Heart rate (bpm)	75±14	64±11	70±13	67±12	<0.001
Mean right atrial pressure (mmHg)	10±4	5±3	8±3	5±3	<0.001
Right ventricular end-diastolic pressure (mmHg)	11±4	7±3	10±4	7±3	<0.001
Systolic pulmonary artery pressure (mmHg)	55±14	35±11	46±14	31±8	<0.001
Diastolic pulmonary artery pressure (mmHg)	23±7	13±4	19±6	11±4	<0.001

Mean pulmonary artery pressure (mmHg)	36±9	21±4	30±8	19±6	<0.001
Mean pulmonary artery wedge pressure (mmHg)	25±6	12±2	21±5	10±3	<0.001
Transpulmonary gradient (mmHg)	11±6	9±4	9±5	9±4	<0.001
Pulmonary vascular resistance (Wood units)	2.6±1.6	2.0±0.9	2.0±1.4	1.8±1.4	<0.001
Pulmonary artery capacitance (ml/mmHg)	2.1±1.3	3.6±1.2	3.1±2.2	4.0±1.6	<0.001
Left ventricular end-diastolic pressure (mmHg) (n=335)	26±7	16±5	24±7	19±7	<0.001
Systemic vascular resistance (Wood units)	21.3±6.0	19.1±4.1	20.5±4.7	19.5±4.5	0.005
Arterial oxygen saturation (%)	95 (93-96)	94 (93-96)	95 (94-97)	96 (94-97)	0.002
Mixed venous oxygen saturation (%)	64 (57-69)	68 (63-73)	67 (63-71)	70 (67-73)	<0.001
Cardiac output (l/min)	4.2±1.1	4.8±1.0	4.8±0.8	4.9±0.9	<0.001
Cardiac index (l/min/m^2)	2.3±0.5	2.6±0.5	2.5±0.4	2.6±0.5	<0.001
Stroke volume (ml)	59±19	73±19	71±18	75±17	<0.001
Stroke volume index (ml/m^2)	32±9	39±10	37±9	40±8	<0.001

Data are given as numbers and percentages, mean±standard deviation or median (interquartile range).

E/e' = ratio of peak early mitral inflow velocity to peak early mitral annular velocity.

Table S2. Follow-up data of patients with versus without loop diuretic therapy (LDT) (n=421)

	**LDT** (n=125)	**No LDT** (n=296)	**P value**
Follow-up time post AVR (months)	16±9	16±6	0.33
NYHA class (n=412)			0.007
I	67 (55%)	210 (72%)	
II	43 (35%)	69 (24%)	
III	11 (9%)	10 (3%)	
IV	1 (1%)	1 (1%)	
Exercise capacity (Watt)	107±40 (n=42)	123±45 (n=152)	0.04
Exercise capacity (%predicted)	89±27 (n=45)	96±24 (n=151)	0.09
Left ventricular end-diastolic diameter (mm)	48±7 (n=95)	48±8 (n=238)	0.53
Left ventricular ejection fraction (%)	59±9 (n=118)	61±9 (n=281)	0.06
E/e'	17±8 (n=38)	14±7 (n=106)	0.01
Left atrial area (cm^2)	29±10 (n=46)	24±8 (n=113)	0.003
Mitral regurgitation (n=399)			<0.001
no	39 (32%)	167 (60%)	
mild	71 (59%)	103 (37%)	
moderate	10 (8%)	8 (5%)	
severe	1 (1%)	0	
Mean aortic valve gradient (mmHg)	11±5 (n=111)	11±4 (n=270)	0.99
Estimated systolic pulmonary artery pressure (mmHg)	37±11 (n=61)	32±8 (n=150)	<0.001
Tricuspid annular plane systolic excursion (mm)	20±5 (n=36)	18±4 (n=71)	0.08

Data are given as numbers and percentages, mean±standard deviation or median (interquartile range).

Dimensions are not indexed to body surface area because height and weight at follow-up were not always available.

E/e' = ratio of peak early mitral inflow velocity to peak early mitral annular velocity.

Supplemental Table S3. Univariate and multivariate Cox regression analysis for the prediction of long-term mortality after aortic valve replacement

	Univariate analysis		Multivariate analysis	
	**HR (95%CI)**	**P value**	**HR (95%CI)**	**P value**
Torasemide dose (per 5 mg)	1.05 (1.01-1.09)	0.01		
Age (per 5 years)	1.21 (1.02-1.44)	0.03		
Body mass index (per kg/m^2)	0.93 (0.87-0.99)	0.03		
Chronic obstructive pulmonary disease	3.01 (1.58-5.91)	0.001	2.97 (1.53-5.76)	0.001
Previous percutaneous coronary intervention	2.58 (1.24-5.36)	0.01	2.31 (1.11-4.81)	0.02
Previous coronary artery bypass grafting	2.77 (1.17-6.55)	0.02		
Oral anticoagulation	2.53 (1.37-4.65)	0.003		
eGFR (per 5 ml/min/1.73 m^2)	0.91 (0.85-0.98)	0.02		
STS score (per ln increase)	2.73 (1.78-4.18)	<0.001	2.78 (1.77-4.35)	<0.001
logistic Euroscore (per ln increase)	2.53 (1.76-3.66)	<0.001		
Left ventricular ejection fraction (per 5%)	0.83 (0.75-0.92)	<0.001		
Mean aortic valve gradient (per 5 mmHg)	0.88 (0.80-0.97)	0.007		
Mitral regurgitation		<0.001		
mild versus no	2.26 (1.10-4.56)	0.03		
moderate versus no	5.42 (2.25-13.09)	<0.001		
severe versus no	7.83 (2.18-28.12)	0.002		

eGFR = estimated glomerular filtration rate; HR = hazard ratio; STS = Society of Thoracic Surgeons; 95%CI = 95% confidence interval.

Supplemental Figure S1. Kaplan Meier plots (cumulative hazard) comparing survival of patients with high-dose loop diuretic therapy (LDT; red), low-dose LDT (green) and no LDT (blue).

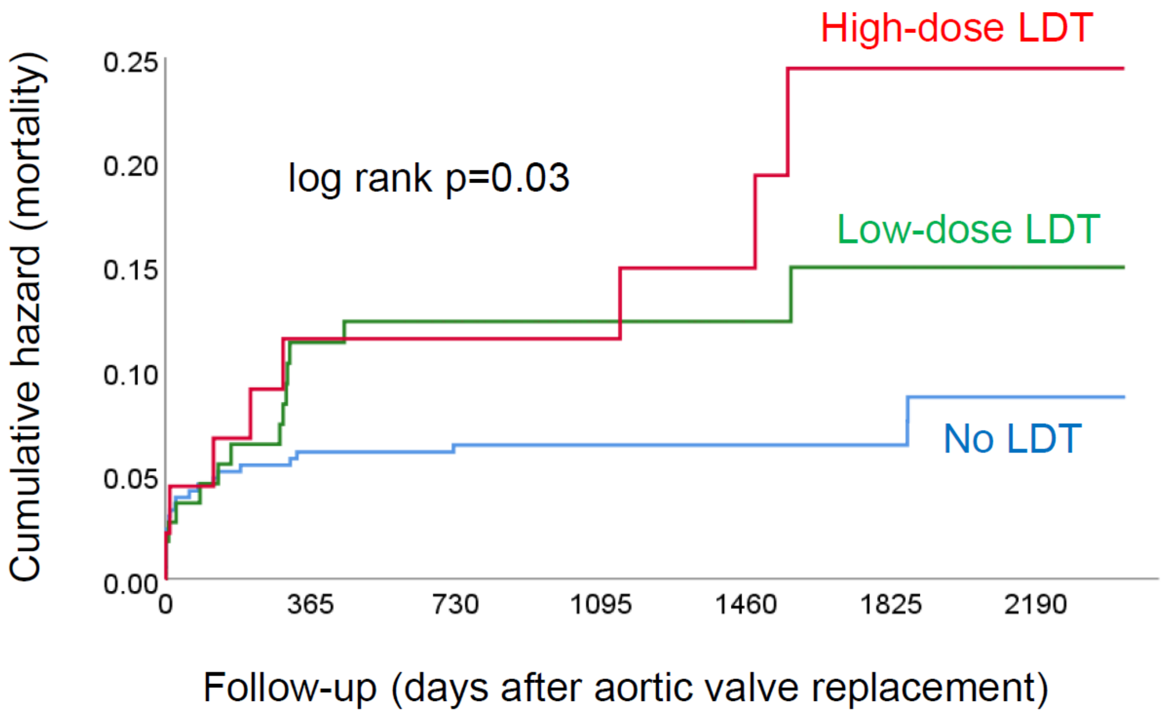

Supplemental Figure S2. Kaplan Meier plots (cumulative hazard) comparing survival of patients in different loop diuretic use (LDT) and pulmonary artery wedge pressure (PAWP) strata. For definitions please see text.

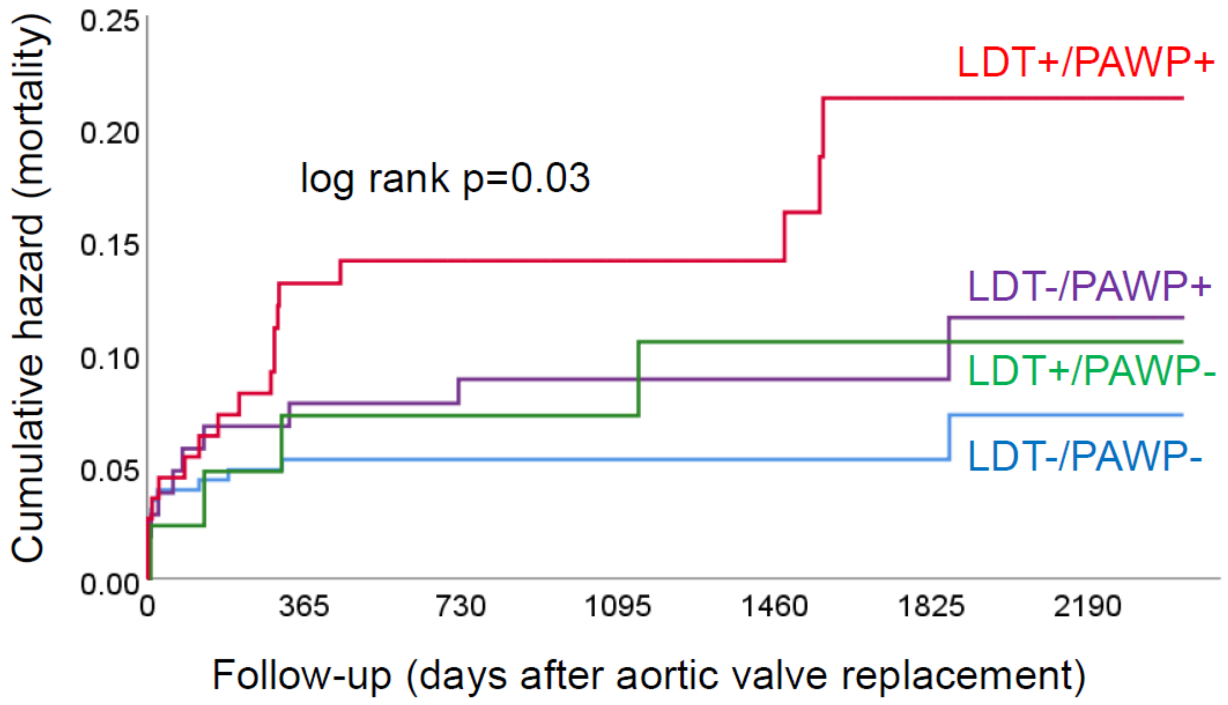

Supplement: Supplementary file 1 — Supplemental Table S1: Data from echocardiography and cardiac catheterization of patients in different loop diuretic therapy (LDT) and mean pulmonary artery wedge pressure (PAWP) strata. For definitions please see text. Table S2: Follow‐up data of patients with versus without loop diuretic therapy (LDT) (n = 421). Supplemental Table S3: Univariate and multivariate Cox regression analysis for the prediction of long‐term mortality after aortic valve replacement. Supplemental Figure S1: Kaplan Meier plots (cumulative hazard) comparing survival of patients with high‐dose loop diuretic therapy (LDT; red), low‐dose LDT (green) and no LDT (blue). Supplemental Figure S2: Kaplan Meier plots (cumulative hazard) comparing survival of patients in different loop diuretic use (LDT) and pulmonary artery wedge pressure (PAWP) strata. For definitions please see text. [file CLC-48-e70225-s001.pdf]
